# Population-based study of the incidence of congenital hip dysplasia in preterm infants from the Survey of Neonates in Pomerania (SNiP)

**DOI:** 10.1186/s12887-017-0829-5

**Published:** 2017-03-16

**Authors:** A. E. Lange, J. Lange, Till Ittermann, M. Napp, Paul-Christian Krueger, H. Bahlmann, R. Kasch, M. Heckmann

**Affiliations:** 1 Departments of Paediatrics and Neonatology & Paediatric Intensive Care Medicine, University of Greifswald, Greifswald, Germany; 2 Department of Trauma Surgery, University of Greifswald, Greifswald, Germany; 3 Institute for Community Medicine, Div. SHIP – Clinical Epidemiological Research, University Medicine Greifswald, Greifswald, Germany; 4 Department of Radiology, University of Greifswald, Greifswald, Germany; 5 Department of Orthopaedics and Orthopaedic Surgery, University of Greifswald, Greifswald, Germany; 6 Klinik und Poliklinik für Kinder- und Jugendmedizin, F.-Sauerbruch-Str., 17487 Greifswald, Germany

**Keywords:** Preterm neonate, Ultrasound of the hip, Hip dysplasia, Screening of neonates

## Abstract

**Background:**

Some etiological factors involved in developmental dysplasia of the hip (DDH) occur in the last trimester of pregnancy, which could result in a decreased incidence of DDH in preterm infants. The aim of this study was to compare the incidence of DDH between preterm and term infants.

**Methods:**

Ultrasound of the hip joint was performed in 2,534 term infants and 376 preterm infants within the population-based Survey of Neonates in Pomerania (SNiP) study.

**Results:**

A total of 42 (1.66%) term infants had DDH (Graf type II c, 0.8%; type D, 0.3% left and 0.4% right; type III a, 0.2% left). Eighteen infants had bilateral findings. Hip dysplasia occurred more frequently in female neonates (32/1,182 vs. 10/1,302, *p* < 0.023; 95% CI 0.012–0.022, *χ* ^2^ test). A familial disposition for DDH was found in 169 (6.7%) term infants and 181 (7.1%) infants in the overall population. In preterm infants, dysplasia of the hip was found in only three late preterm infants with gestational age between 36 and 37 weeks (*n* = 97) and not in preterm infants <36 weeks gestational age (*n* = 279). Regression analysis revealed a narrowly significant association between gestational week of birth and DDH (relative risk = 1.17; 95% confidence interval 0.99–1.37; *p* = 0.065).

**Conclusion:**

Our study suggests that preterm infants <36 weeks gestational age have a decreased risk of DDH.

## Background

Developmental dysplasia of the hip (DDH) is one of the most common congenital malformations with a regionally varying incidence of 1.5–4.9% [1–5]. The diagnostic standard for DDH is ultrasound imaging using the Graf classification [6–8]. In 1996, as part of the guidelines for early detection of childhood disease, Germany implemented screening for hip dysplasia in the form of a routine hip ultrasound for all neonates at 4–6 weeks of life. Many hospitals deviate from this by performing the hip ultrasound at 3–10 days of life as part of the routine examination (U2) given at this time. Schilt et al. screened nearly 15,000 babies immediately after birth over 15 years and showed that earlier treatment may lead to a shorter duration [1, 2]. Preterm infants are generally screened at a corrected age of more than 36 weeks [9].

Hip development is based on the interaction between genetically determined maturation of the cartilage and bone elements of the acetabulum and the pressure applied by the muscular forces of the centered femur head [10]. Regarding etiology, there are endogenous, exogenous, and genetic factors that cause malposition of the femur, which in turn causes malformation of the acetabulum or too shallow of an acetabulum to form with primary malpositioning of the femur head [10]. Known risk factors for DDH are breech presentation, oligohydramnion, or multiparous pregnancies, resulting in less intrauterine space. In addition, skeletal malformations and neuromuscular disease due to teratogenic agents can also cause types of hip luxation [11]. Furthermore, gender plays a role, with a two- to three-times higher incidence of DDH in females compared to males [12].

Only a few studies have reported the incidence of hip dysplasia in preterm infants, which has been reported to be 0.2–2.8 [11–13] compared to 1.5–4.9 in term infants [1–3].

Intrauterine risk factors for DDH with respect to less intrauterine space occur in the last trimester and are partially or completely absent in the case of preterm birth. The objective was to test the hypothesis that the incidence of DDH is lower in preterm infants than term infants by comparing the incidence of hip dysplasia between these groups. For this purpose, the population-based cohort study Survey of Neonates in Pomerania (SNiP) served as the database [14].

## Methods

### Survey of Neonates in Pomerania

The SNiP study is a prospective, population-based survey that collected comprehensive data on pregnancies, mothers, and their offspring in eastern Pomerania. Details were reported elsewhere [14]. All neonates whose place of residence was in eastern Pomerania were eligible for this survey. Infants were excluded from the survey if communication was impaired due to language barriers or a consent form was missing.

Trained physicians participating in the survey according to a standardized protocol collected the data. The questionnaires were pseudonymized and entered into the study database by a medical archivist.

In the study, data for 95% of all pregnant women in the Ostvorpommern region were recorded and >80% of mothers actively participated over the years. For the non-participants, risk factors during pregnancy, birth mode, and outcome of the child were recorded.

SNiP received a positive vote from the Ethics Committee of the University Medicine Greifswald and written informed consent was obtained from the parents.

### Gestational age

Gestational age was defined as the time elapsed during the pregnancy from the first day of the woman’s last menstrual cycle to the current date. A normal pregnancy ranges from 37 to 42 weeks. To investigate the influence of gestational age on hip development, preterm infants were categorized as late preterm (34 weeks and 0 days–36 weeks and 6 days gestation), very preterm (28 weeks and 0 days–33 weeks and 6 days gestation), or extremely preterm (less than 28 weeks gestation). To investigate the influence of birth weight, preterm infants were categorized as having normal birth weight (>2,500 g), low birth weight (1,500 g–2,500 g), very low birth weight (1,000 g–1,500 g), or extremely low birth weight (<1,000 g).

### Ultrasound examination

**Fig. 1:**
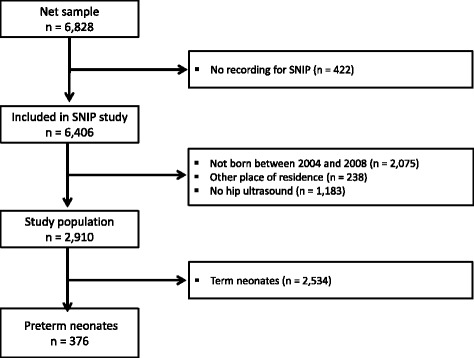
Effect of gestational age and birth weight on Graf classification in preterm infants of the SNiP cohort. For the left (*p* < 0.001) and right hip (*p* < 0.001), extremely preterm and very preterm neonates had a significantly higher incidence of mature hips than late preterm babies. *χ* ^2^ 95% confidence interval 0.99–1.37

Ultrasound of the hip was established for preterm infants not before January 2004. Therefore, only data on hip ultrasound in term and preterm infants born between January 2004 and November 2008 (*n* = 2,910) were included in this subgroup-analysis of the SNiP-cohort. The subgroup represented 42.6% of all live births and 45.4% of all infants included in SNiP between January 2002 and November 2008 (Fig. 1). During this period, 80% of all preterm infants and 70% of term infants underwent a hip ultrasound. Neonates with major congenital malformations, muscular or skeletal disease, or chromosomal anomalies were excluded.

For term infants, ultrasound screening of the hip was performed at the time of the regular second health check (3–10 days of age, called U2 in Germany) in the maternity ward. Briefly, the U2 comprises a physical exam, medical history, and counseling the mother on preventive measures, such as Vitamin D and K prophylaxis and vaccination. Preterm infants were screened at the corrected age of more than 36 weeks. Ultrasound scans, documentation, and classification were performed according to Graf [6–8]. The standard protocol was described previously by Graf [7].

A familial disposition for DDH was defined as dysplasia of the hip in first degree family members (siblings, parents, grandparents), or family members with hip replacement before 50 years of age.

All examiners passed their specialist’s examination as a pediatrician. The structured training comprised a minimum number (*n* = 200) of performed hip ultrasounds. The final board certification implied an assessment of the candidate’s ability to perform an ultrasound. In addition, a DEGUM II certified examiner supervised all pathological results (DEGUM: Deutsche Gesellschaft für Ultraschall in der Medizin). All research was conducted in a Graf storage tray [7] and an ultrasonic instrument with a 7.5 MHz linear transducer (Logia TM 200, Pro Serius, 7, 5-MHz linear transducer, Wi Pro GE Medical Systems, Bangalore, India).

### Statistical analysis

Continuous data were expressed as mean and standard deviation according to their normal distribution and categorical data as absolute numbers and percentages. Differences in mature hip prevalence across groups of gestational age and birth weight were tested by Fisher’s exact test. We also performed a Poisson regression to test whether gestational week of birth was significantly associated with the prevalence of hip dysplasia. A *p*-value < 0.05 was considered significant. A medical statistician (T.I.) carried out all analyses using Stata 13.1 (Stata Corporation, College Station, TX, USA).

## Results

**Fig. 2:**
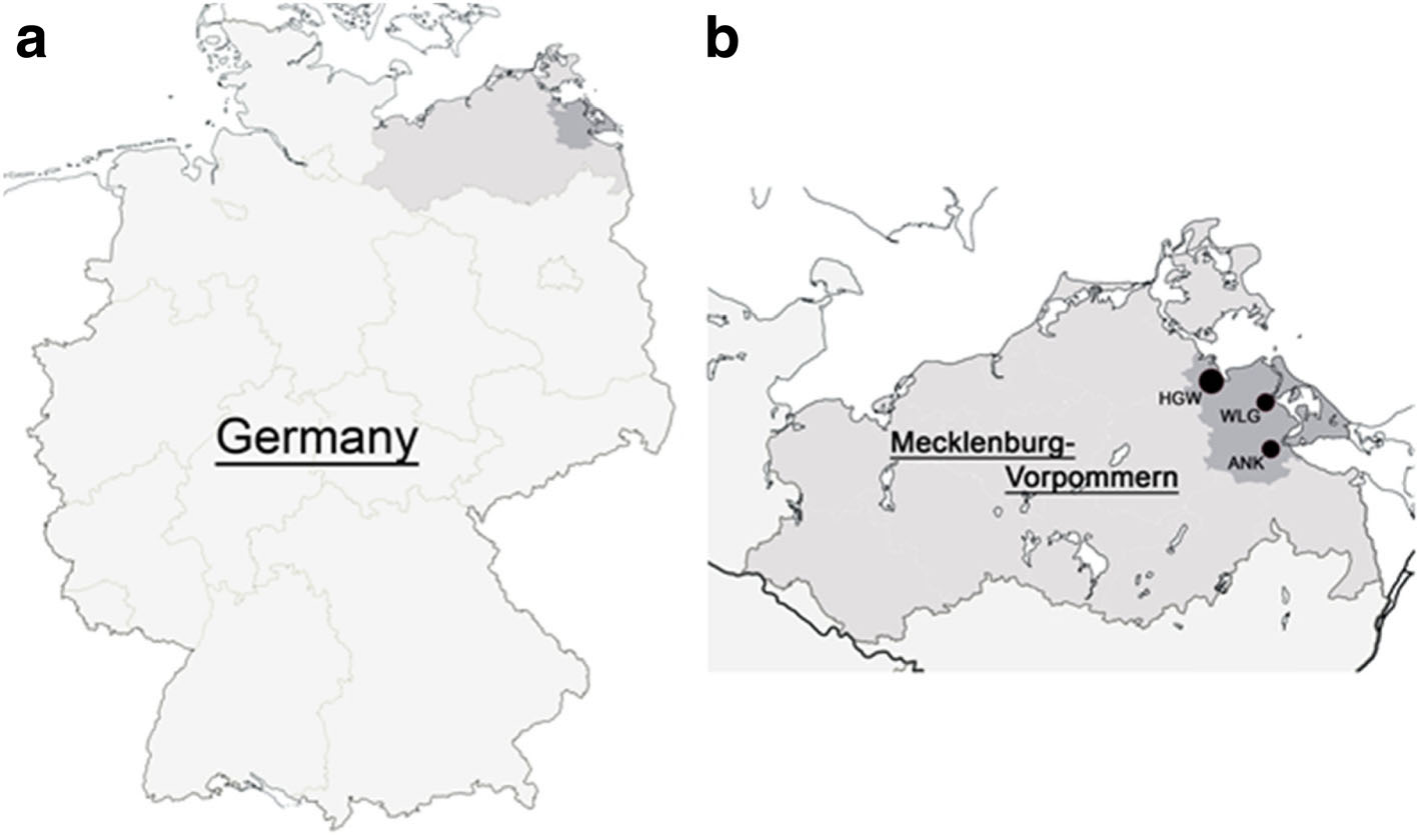
Characteristics of the SNiP population. Ultrasound examination of the hip was established in January 2004 and was available in 2,910 infants born between then and November 2008. This subpopulation represented 80% of all preterm infants and 70% of all term infants included in SNiP during the time period. The study region of the Survey of Neonates in Pomerania (SNiP) was ‘Ostvorpommern’ (**a**, dark grey). SNiP was carried out in three hospitals located in the Hansestadt Greifswald (HGW), Wolgast (WLG) and Anklam (ANK) (**b**)

Hip ultrasound was documented in 2,534 term neonates and 376 preterm neonates born between January 2004 and November 2008 (Fig. 2). The perinatal characteristics of the study population are shown in Table 1. Preterm infants were more often twins (*p* < 0.001) and more often born in breech presentation than term infants (*p* < 0.001). A familial disposition for hip disease did not differ between term and preterm neonates. Table 2 shows the Graf classification of the hips in term and preterm infants.

**Table 1:** Characteristics of the study population

	Term neonates	Preterm neonates
	*N* = 2534	*N* = 376
Gestational age (w)	39.4 (1.2)	33.0 (3.4)
- Late Preterm (34 weeks–36 weeks and 6 days)	-	223 (59.2%)
- Very preterm (28 weeks–33 weeks and 6 days)	-	115 (30.7%)
- Extremely Preterm (<28 weeks)	-	38 (10.1%)
Birth weight (g)	3,463 (480)	2,067 (724)
- Normal birth weight (≥2,500 g)	2,473 (97.6%)	121 (32.1%)
- Low birth weight (<2,500 g)	63 (2.5%)	168 (44.8%)
- Very low birth weight (<1,500 g)	0 (0.0%)	41 (10.9%)
- Extremely low birth weight (<1,000 g)	0 (0.0%)	47 (12.5%)
- SGA	33 (1,5%)	78 (21,4%)
- IUGR	1 (0.03%)	5 (1.3%)
Gender		
- Female	1,211 (47.8%)	183 (48.4%)
- Male	1,320 (52.1%)	193 (51.1%)
Parity		
- Twin	28 (1.1%)	67 (17.7%)
- Triplet	0 (0.0%)	4 (1.1%)
Delivery presentation		
- Cephalic	2,299 (90.7%)	285 (75.8%)
- Breech	152 (6.1%)	78 (20.7%)
Familiary Disposition for developmental dysplasia of the hip	169 (6.7%)	12 (3.1%)

Data are presented as mean (standard deviation) or absolute numbers (percentage). *SGA* small for gestational age (defined as birthweight < 3th.percentile *

*IUGR* intrauterine growth retardation (defined as birthweight, birth length, <3th percentile)

*[New percentile values for the anthropometric dimensions of singleton neonates: analysis of perinatal survey data of 2007–2011 from all 16 states of Germany]

Voigt M, Rochow N, Schneider KT, Hagenah HP, Scholz R, Hesse V, Wittwer-Backofen U, Straube S, Olbertz D

Z Geburtshilfe Neonatol. 2014 Oct;218 (5):210–7. doi:10.1055/s-0034-1385857. German. PMID:25353215

**Table 2:** Graf classification in term and preterm infants of the birth cohort

	Term (*n* = 2,534)		Preterm (*n* = 376)	
Type	Left hip	Right hip	Left hip	Right hip
I a	1,449 (57.2%)	1,462 (57.7%)	296 (78.7%)	302 (80.3%)
II a	1,052 (41.5%)	1,041 (41.1%)	78 (20.5%)	72 (18.9%)
II c	21 (0.8%)	20 (0.8%)	1 (0.3%)	0 (0.0%)
D	8 (0.3%)	9 (0.4%)	1 (0.3%)	2 (0.5%)
III a	2 (0.2%)	0 (0.0%)	0 (0.0%)	0 (0.0%)
IV	0 (0.0%)	0 (0.0%)	0 (0.0%)	0 (0.0%)

Data are presented as absolute numbers (percentage)

Survey of Neonates in Pomerania (SNiP)

For the left (*p* < 0.001) and right hip (*p* < 0.001), preterm infants had a significantly higher incidence of mature hips than term infants. The incidence of Graf II was twice as high in term infants as in preterm infants. Overall, the incidence of Graf II c or higher dysplasia of the hip was greater in term infants (left hip: 1.3%, right hip: 1.2%) than in preterm infants (left hip: 0.53%, right hip: 0.53%). By individual years, the incidence of hip dysplasia in term infants was 2.10% in 2004, 2.23% in 2005, 2.2% in 2006, 0.9% in 2007, and 1.5% in 2008. A familial disposition for dysplasia of the hip was found in 169 (6.7%) term infants and 181 (7.1%) infants in the overall population.

**Fig. 3:**
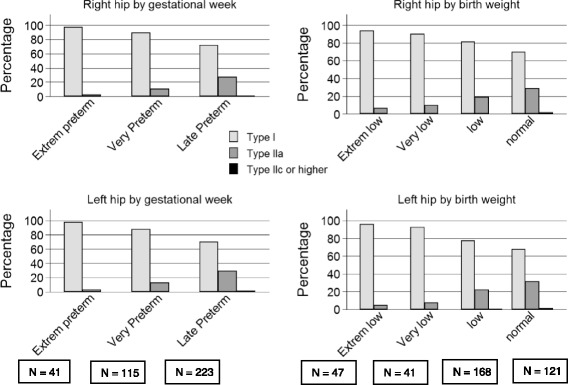
Gestational age and risk of developmental dysplasia of the hip (DDH). Regression analysis of the association between gestational week of birth and risk of DDH (relative risk = 1.17, 95% confidence interval 0.99–1.37, *p* = 0.065). One week of gestation was associated with a 16% increased risk of hip dysplasia. Dotted line: 95% confidence interval 1.08–1.63

Figure 1 shows the influence of gestational age and birth weight on Graf classification in preterm infants. For the left (*p* < 0.001) and right hip (*p* < 0.001), extremely preterm and very preterm neonates had a significantly higher incidence of mature hips than late preterm babies (*χ* ^2^ test). The incidence of Graf II a was 10-times higher in late preterm infants than in extremely preterm infants, and three-times higher than in very preterm neonates. Only three late preterm infants with a gestational age of more than 36 weeks had dysplasia of the hip: two female preterm neonates (the first born 36 weeks and 4 days, 2,520 g, transverse position, right and left hip type D; the second one was born 36 weeks, 2,480 g, breech position, right hip type II a, left hip type II c) and one male preterm neonate (born 36 weeks and 1 day, 2,700 g, cephalic position, right hip type D, left hip type II a). Preterm infants with extremely low or very low birth weight had a higher incidence of mature hips than preterm babies with low or normal birth weight for the left (*p* < 0.001) and right hip (*p* = 0.004). The incidence of type II a was six-times higher in preterm infants of normal birth weight than preterm infants with extremely low birth weight. In the Poisson regression, we found no significant differences between term infants (1.75%) and preterm infants (0.53%) with respect to the prevalence of hip dysplasia (relative risk = 2.14, 95% CI 0.66–6.92, *p* = 204). Therefore, we added analyses based on the continuous gestational week of birth variable, which revealed a barely significant association between gestational week of birth and dysplasia of the hip (relative risk = 1.17, 95% CI 0.99–1.37, *p* = 0.065). One week of gestation was associated with a 16% increased risk of hip dysplasia (Fig. 3).

## Discussion

Intrauterine risk factors for DDH in the last trimester are partially or completely absent in the case of preterm birth. In our large cohort of preterm infants, hip dysplasia was not found at less than 36 weeks gestational age. Our findings are consistent with recently published long-term studies conducted in infants during the neonatal period to evaluate multiple risk factors for DDH. In our preterm cohort, the incidence of dysplasia of the hip defined as type II c or greater was 0.53%. Compared to unselected newborns, preterm babies exhibited a significantly higher incidence of types I a and II a.

In contrast, the incidence of DDH in our term group corresponds to the findings in the literature [1–3]. Further findings in our term group are in accordance with the literature, including female neonates being affected two- to three-times more often than their male counterparts [11]. In our study, 32 female and 10 male neonates had hip dysplasia. Moreover, the left hip is more often affected because of the high prevalence of cephalic vertex presentation at birth [15]. In over 94% of neonates who are born from the cephalic presentation the fetal lie is the left occipito anterior position. In this position, the back of the fetus is left and the left hip lies narrowed to the mother’s spine. Therefore, it could be speculated that the left hip joint is more likely to be restricted in mobility than the right one [16].

In our cohort, the incidence of fully mature hip joints was distinctly higher in preterm neonates than in term neonates (Table 2). At the time of ultrasound screening, 80% of preterm neonates had a Graf I a hip classification and approximately 20% still had a physiological maturation deficit (Graf II a), whereas 41% of term neonates were classified as type II a. Bick et al. examined the hips of 92 preterm neonates by ultrasound over the period of 1 year. Using the Graf classification, only 7% had an angle alpha between 50 and 60°, which is characteristic of type II a hips. In all other examinations, the angle alpha was > 60° (type I). Sonographically there were no pathological findings [15]. Timmler et al. showed in a large cohort study comprising 143 preterm infants that preterm delivery was not a risk factor for the development hip dysplasia [5].

Sezar et al. showed that prematurity regardless of maternal risk factors did not have an effect on the incidence of DDH [17]. Preterm infants are no longer subject to maternal hormones or lack of intrauterine space after their birth and have complete freedom to move their lower extremities. Due to better cardiorespiratory stability, the prone position is preferred in preterm infants [18]. If these infants are placed on their backs, thick diapers encourage correct positioning of the femur head in the acetabulum. All of these factors should be discussed as stimuli for development of the hip joint. These preterm infants are not subject to intrauterine restrictions; therefore, muscular, cartilaginous, and bone development can occur unhindered. In addition, maternal steroid hormones lead not only to the relaxation of maternal ligaments, but also similar effects on the ligaments of the fetal hip joint [19, 20]. These suggestions are supported by our finding that the proportion of mature hips was greater in the group of the most immature infants (Fig. 1).

### Strengths and limitations

Our study has several limitations. First, we report on data from a birth cohort study that was not designed to exclusively investigate hip ultrasound data. Second, the examiners were not particularly trained or re-evaluated for the study; therefore, intra- and inter-observer variability could not be reported. Nevertheless, high quality standards for qualification of the examiners were applied. Third, our study did not report on outcome data (re-examination of the immature hips and details of therapy), which may have influenced the results due to the low incidence of DDH. However, the strengths of this study were the large number of newborns, particularly preterm infants, investigated and that the cohort was population-based.

## Conclusion

Our study suggests that preterm infants (<36 weeks gestational age) have a decreased risk of DDH. Furthermore, the incidence of fully mature hip joints was twice as high in preterm infants as in term infants and the proportion of mature hips was highest in the group with the most immature infants. These findings suggest that the absence of intra-uterine factors, such as lack of space, atypical positioning (e.g., breech presentation), and maternal steroid hormones, promotes maturation of the hip in preterm infants.

## References

[1] Partenheimer A, Scheler-Hofmann M, Lange J (2006). Correlation between sex, intrauterine position and familial predisposition and neonatal hip ultrasound results. Ultraschall Med.

[2] Schilt M (2001). Optimal age for hip sonography screening. Ultraschall Med.

[3] Exner GU, Mieth D (1987). Sonographic screening for hip dysplasia in newborn infants. Schweiz Med Wochenschr.

[4] Treiber M (2008). Ultrasound screening for developmental dysplasia of the hip in the newborn: a population-based study in the Maribor region, 1997–2005. Wien Klin Wochenschr.

[5] Timmler T (2005). The hip joints of preterm neonates in sonographic evaluation. Chir Narzadow Ruchu Ortop Pol.

[6] Graf R (1984). Fundamentals of sonographic diagnosis of infant hip dysplasia. J Pediatr Orthop.

[7] Graf R (1985). Hip sonography in infancy. Procedure and clinical significance. Fortschr Med.

[8] Graf R (1982). The anatomical structures of the infantile hip and its sonographic representation. Morphol Med.

[9] Tuncay IC (2005). Is prematurity important in ultrasonographic hip typing?. J Pediatr Orthop B.

[10] Dezateux C, Rosendahl K (2007). Developmental dysplasia of the hip. Lancet.

[11] Stevenson DA (2009). Familial predisposition to developmental dysplasia of the hip. J Pediatr Orthop.

[12] Fox AE, Patron RW (2010). The relationchip between mode of delivery and developmental dysplasia of the hip in breech infants: a 4-year prospective cohort study. J Bone Joint Surg (Br).

[13] Yau CH (2012). Frequency of developmental dysplasia of the hip in breech-presented Chinese neonates in Hong Kong. Hong Kong Med J.

[14] Ebner A, Thyrian JR, Lange A, Lingnau ML, Scheler-Hofmann M, Rosskopf D (2010). Survey of Neonates in Pomerania (SNiP): a population-based birth study--objectives, design and population coverage. Paediatr Perinat Epidemiol.

[15] Bick U, Muller-Leisse C, Troger J (1990). Ultrasonography of the hip in preterm neonates. Pediatr Radiol.

[16] Quan T, Kent AL, Carlisle H (2013). Breech preterm infants are at risk of developmental dysplasia of the hip. J Paediatr Child Health.

[17] Sezer C, Unlu S, Demirkale I (2013). Prevalence of developmental dysplasia of the hip in preterm infants with maternal risk factors. J Child Orthop.

[18] van der Burg PS, Miedema M, DeJongh FH, Frerichs I, Van Kaam AH (2015). Changes in lung volume and ventilation following transition from invasive to noninvasive respiratory support and prone positioning in preterm infants. Pediatr Res.

[19] Ishikawa N (2008). The relationship between neonatal developmental dysplasia of the hip and maternal hyperthyroidism. J Pediatr Orthop.

[20] Trotter A (1999). Effects of postnatal estradiol and progesterone replacement in extremely preterm infants. J Clin Endocrinol Metab.

